# Hearing Loss and Default‐Mode Network Connectivity in Older Adults with Subjective Cognitive Decline and Mild Cognitive Impairment

**DOI:** 10.1002/alz.091882

**Published:** 2025-01-09

**Authors:** Nicole Grant, Natalie Phillips

**Affiliations:** ^1^ Concordia University, Montreal, QC Canada

## Abstract

**Background:**

Hearing loss (HL) is a risk factor for dementia that is prevalent in older adults. Altered brain connectivity is present in individuals with HL, individuals with subjective cognitive decline (SCD), and individuals with mild cognitive impairment (MCI). In individuals with HL, altered brain connectivity has been associated with impaired scores on cognitive tests. In individuals with MCI, altered brain connectivity has been associated with degree of cognitive impairment and progression to dementia. Therefore, altered brain connectivity is a potential mechanism by which HL is associated with increased dementia risk.

**Methods:**

Data are from the Comprehensive Assessment of Neurodegeneration in Dementia study (Data Release 6, May 2023). Participants include cognitively unimpaired (CU, n=78, age=69.5), %female=77%), SCD (n=85, age=70.0, %female=74%), and MCI (n=159, age=72.2), %female=40%) individuals. Independent component analysis was used to identify the default‐mode network (DMN), a resting‐state network that is altered in individuals at risk for dementia. Hearing data include a pure‐tone hearing screening protocol and speech‐in‐noise perception. Based on the pure‐tone hearing screening, participants were classified as having normal hearing (NH) or HL. In each diagnostic group (CU, SCD, MCI), analyses of covariance compared DMN connectivity between the NH and HL participants and linear regressions evaluated the relationship between speech‐in‐noise perception and DMN connectivity.

**Results:**

Preliminary results are available for 94 older adults with MCI (NH = 60, pure‐tone HL=34). Compared to individuals with MCI and NH, those with MCI and HL had decreased connectivity between the DMN and the caudate and thalamus. There was no relationship between DMN connectivity and speech‐in‐noise perception. The CU and SCI groups are expected to have a greater capacity for compensatory changes in functional connectivity. Therefore, it is expected that in the CU and SCI groups, pure‐tone HL and speech‐in‐noise perception will be associated with increased DMN connectivity.

**Conclusion:**

Current results suggest that the increased dementia risk associated with HL may be due to decreased DMN connectivity and that pure‐tone HL and speech‐in‐noise perception are differentially related to DMN connectivity. Future research will determine whether the relationship between hearing and DMN connectivity differs between older adults with varying risk of cognitive impairment.

